# Self-management interventions for skin care in people with a spinal cord injury: part 2—a systematic review of use of theory and quality of intervention reporting

**DOI:** 10.1038/s41393-018-0136-5

**Published:** 2018-05-25

**Authors:** Justine S. Baron, Katrina J. Sullivan, Jillian M. Swaine, Arlene Aspinall, Susan Jaglal, Justin Presseau, Dalton Wolfe, Jeremy M. Grimshaw

**Affiliations:** 1 Clinical Epidemiology Program, Ottawa Hospital Research Institute, Ottawa, ON Canada; 2 Faculty of Health and Medical Sciences, University of Western Australia, Perth, WA Australia; 3 Institute for Health Research, University of Notre Dame Australia, Fremantle, WA Australia; 4 Fiona Stanley Hospital, State Rehabilitation Service, Spinal Service, Perth, WA Australia; 5 Rick Hansen Institute, Vancouver, BC Canada; 6 Vancouver General Hospital, Vancouver, BC Canada; 7 Department of Physical Therapy, University of Toronto, Toronto, ON Canada; 8 Toronto Rehabilitation Institute, Toronto, ON Canada; 9 School of Epidemiology and Public Health, University of Ottawa, Ottawa, ON Canada; 10 Parkwood Institute Research, Lawson Health Research Institute, London, ON Canada; 11 University of Western Ontario, London, ON Canada; 12 Department of Medicine, University of Ottawa, Ottawa, ON Canada

## Abstract

**Study design:**

Systematic review.

**Objectives:**

To examine use of theory and quality of reporting in skin care self-management interventions for people with SCI.

**Setting:**

International.

**Methods:**

The Theory Coding Scheme (TCS) and the Template for Intervention Description and Replication (TIDieR) checklist were applied by two independent researchers to 17 interventions identified in a systematic review of self-management interventions for skin care in people with SCI.

**Results:**

Six (35%) of the 17 interventions reviewed were reported to have a theoretical basis. Theories used included three of the most commonly featured in health behavior research (the Health Belief Model, Social Cognitive Theory, and the Transtheoretical Model). In these six interventions, theory was used to design content but not to select participants or tailor strategies. None of the interventions were used to test theories in the SCI population, or to propose theoretical refinements. Reporting quality was found to vary by TIDieR item, with 6–100% of interventions including recommended information. Information on two intervention fidelity items was missing in 53 and 82% of descriptions.

**Conclusions:**

Use of theory and reporting quality in SCI self-management research remains suboptimal, potentially slowing down advancements in this area of research. Rehabilitation researchers should direct their efforts toward improving these practices to help build a science of SCI self-management that is cumulative and reproducible by clinicians, scientists, and policy makers.

**Sponsorship:**

This work was funded through a postdoctoral fellowship awarded to the first author by the Rick Hansen Institute.

## Introduction

Pressure ulcers (or injuries) are one of the commonest and most challenging clinical problems people with a spinal cord injury (SCI) experience. They can result in recurrent hospitalizations, surgeries, and are life-threatening in end-stage cases [1]. They have become a health care priority in recent years, particularly now that most pressure ulcers in clinical settings are considered avoidable with appropriate care [2]. Following their time in acute and rehabilitation settings, newly injured people with SCI discharged back to the community are required to engage in preventative skin care behaviors similar to those adopted in clinical settings (e.g., skin checks, pressure relief, protein intake) [3]. Research, however, suggests that adherence to these SCI skin care recommendations is suboptimal [4, 5]. Consequently, a growing number of self-management interventions have been designed to equip people with a SCI with the necessary knowledge, skills, and confidence to engage in these preventative skin care behaviors. Despite these efforts, a trend toward an increase in pressure ulcer prevalence in community-dwelling people with SCI has been observed [6].

In light of the pressing problem that pressure ulcers pose in the SCI population and the severity of their consequences, the need for the SCI self-management researchers to build a solid evidence base in a timely manner is reinforced. One concern that jeopardizes this is that rehabilitation and self-management evaluations are often considered to be a “black box” [7, 8]. In science, this labeling usually refers to two main problems. The first problem is that researchers do not always explicitly report the theories on which the interventions and evaluations they design are based [9]. Although this could be the result of poor reporting practices, it is likely to reflect actual lack of theory use at the study design stage. Interventions designed without theoretical considerations may be unclear about the specific behavior(s) or outcomes they target, the processes they aim to change, the mechanism through which anticipated effects may occur, or the methods used to assess this impact [10]. Failure to use theory therefore impedes the efficacy and understanding of these interventions and limits the learning that can be used in future intervention planning [10]. The second problem that results in the “black box” labeling is the poor reporting of treatment descriptions. Incomplete reporting of intervention descriptions affects the building of an evidence base by making difficult the interpretation and replication of study results [11]. Researchers cannot interpret or reproduce interventions on which essential information is missing or unclear (e.g., unclear about the active ingredients delivered, mode(s) of delivery, timing, dosage, location, intervention providers). Collecting and reporting data on intervention fidelity is part of the accurate description of tested interventions [11]. Modifications of intervention protocols during implementation need to be described with transparency for correct interpretation of outcomes and replication to be possible.

Shortcomings in theory use and intervention reporting are not specific to rehabilitation and self-management sciences [9]. Their detrimental effect on the building of a solid evidence base is likely amplified by the inherent complexity of the interventions delivered in these areas of work. Given these concerns and the pressing need for effective SCI skin care self-management interventions, our program of work aims to examine the existing evidence base on these interventions. A first project consisted in a systematic review to investigate skin care self-management interventions (see published protocol [12]). Results of this systematic review are reported in two publications. A first publication details findings on the content and effectiveness of these interventions [13]. This second publication reports on the use of theory and quality of intervention reporting in these interventions. By assessing the state of these research practices in SCI skin care self-management research, our aim is to highlight practice gaps that, if addressed, can strengthen the available evidence base.

## Methods

### Search strategy

Electronic bibliographic databases: Search strategies that included terms relating to SCI, self-management, and skin care were designed for nine electronic bibliographic databases (MEDLINE, Embase, PsycINFO, CENTRAL, CINAHL, REHABDATA, CIRRIE, PeDro, ERIC) and reviewed by a librarian for comprehensiveness and accuracy. They were run on 23 February 2016.

Additional data sources**:** JSB searched for relevant papers using (1) posters, abstracts, and conference proceedings identified in electronic bibliographic database search results, (2) hand-searching of reference lists in relevant protocols, systematic reviews, and of eligible studies, (3) keyword searches in electronic prospective trial registers on 21 June 2016 (World Health Organization International Clinical Trials Registry, and the Meta-Register of Controlled Trials). Authors or principal investigators of relevant studies were contacted to identify relevant published or forthcoming publications.

Search strategies applied to electronic databases are presented in Supplementary Information 1.

### Eligibility criteria

As per protocol [12], eligibility criteria listed in Table 1 were defined to address a series of research questions including theory use and intervention reporting quality. Requirements relating to measurement of outcomes of interest were used to help determine whether an intervention was sufficiently skin care oriented for inclusion in this review.

**Table 1:** Eligibility criteria

Inclusion criteria	
Publication status	Published papers only
Publication date	No restrictions
Language	English
Study design	Randomized controlled trials and non-randomized trials
Control or comparison	At least one control group No restrictions on number of intervention groups
Population	At least 50% of participants diagnosed with a spinal cord injury
Intervention	Interventions aiming to improve, at least in part, self-management skin care capabilities related to pressure ulcer prevention
Intervention setting	No restrictions
Outcome	Measurement of at least one outcome of interest: mediators of skin care behaviors (e.g., knowledge, self-efficacy, or skills in relation to skin care or pressure ulcer prevention etc.), skin care behaviors (e.g., skin checks, pressure relief), and pressure ulcer-related clinical outcomes (e.g., incidence, reoccurrence, severity, or hopsitalization)
Length of follow-up	No restrictions
Exclusion criteria	Interventions with a primary focus on pressure ulcer treatment Interventions with a primary focus on lifestyle-related behaviors that affect physiological indicators of skin health (e.g., improving nutritional intake or physical activity, smoking cessation)

### Study selection

Two-level screening (first titles and abstract, then full texts) of bibliographic database search results was performed by two independent reviewers (JSB and JMS). Publications identified in additional data sources by JSB and considered to have relevant titles and abstracts were referred to the second reviewer (JMS) for same level screening (publications considered irrelevant by JSB were not reviewed by JMS). Full texts of publications approved by JMS for full-text review were then retrieved and screened independently by JSB and JMS. A third reviewer (JMG) was consulted if JSB and JMS could not agree on a screening outcome during any of the above screening processes. Discussions continued until consensus was reached.

### Data extraction

Excel spreadsheets were designed to capture information relevant to items in the theory use and intervention reporting tools described below. Two researchers (JSB and KJS) with experience in health psychology and knowledge translation independently applied these two tools to the interventions described in eligible papers. Published materials linked to eligible studies via personal communications with authors of included papers or in-text references, as well as during reference screening, were used in data extraction. A third party (JG) was consulted if disagreements between the two reviewers during data extraction remained unresolved after discussion.

#### Use of theory

The Theory Coding Scheme (TCS) [14] is a 19 item checklist that allows for an in-depth analysis of *whether* and *how* theory is used in the design and evaluation of behavioral interventions. Each item is coded “Yes” or “No” and the tool has been shown to have good inter-rater reliability [14]. The coding scheme items cover six domains: (1) is theory mentioned?, (2) are the relevant theoretical constructs targeted?, (3) is theory used to select recipients or tailor interventions?, (4) are the relevant theoretical constructs measured?, (5) is theory tested?, and (6) is theory refined?. Following communications with the authors of the TCS, items 13 and 14 (assessing the quality of the measures used and presence of successful randomization methods) were removed as they were not considered relevant to theory use. As per TCS instructions, the TCS was applied using the information provided for each intervention. We used example papers previously reported [15] to explicitly describe links between theory and intervention techniques to guide our application of items 7–11. Diagram explanations to help interpret these items can be found in the original TCS paper [14].

#### Intervention reporting quality

The Template for Intervention Description and Replication (TIDieR) [11] is a reporting guideline that can be used to assess completeness of intervention descriptions. It proposes 12 essential elements to include in the description of a health intervention including brief name of intervention (item 1), why (i.e., rationale, or theory underlying intervention, item 2), what (intervention materials for participants and providers, items 3a and 3b), intervention procedures (item 4), who provided (intervention providers, item 5), how (modes of delivery, item 6a; individual or group, item 6b), where (intervention setting, item 7), when and how much (i.e., number of sessions, item 8a; schedule/frequency, item 8b; duration of sessions, item 8c), tailoring (i.e., what, why, when, and how intervention was personalized, item 9), modifications (changes to protocol, item 10), how well (fidelity assessment methods, item 11a; fidelity promotion strategies, 11b), and extent to which intervention was delivered as planned (actual fidelity, item 12). Each item is coded “No, not fully reported” (no mention or incomplete information in intervention description) or “Yes, fully reported” (complete information available). The decision rules applied are provided in Supplementary Information 2.

## Results

A PRISMA flow diagram is available in Supplementary Information 3. Ten randomized controlled trials and five non-randomized trials testing 17 interventions were identified to meet the inclusion criteria. Interventions (*k*) consisted of structured educational programs (*k* = 5) [16–21], telehealth (*k* = 6) [22–26], wheelchair skills training (*k* = 3) [27–29], risk assessment and feedback (*k* = 2) [30, 31], and body positioning training (*k* = 1) [32]. They were delivered across a range of settings (e.g., inpatient, outpatient, community settings) and varied in length and the extent of their focus on skin care. A detailed description of study characteristics is not within the scope of this publication, but is available elsewhere [13]. Scientific publications linked to primary papers and used for data extraction included papers on intervention development/content [33, 34], protocol [35], pilot test [36], and an erratum [16].

### Use of theory

Table 2 illustrates how theory was used in the design and evaluation of the 17 interventions reviewed. Six interventions [19–21, 25, 26, 29, 32] were explicitly reported to have based their intervention on theory (i.e., item five coded “yes”) with four [19, 20, 29, 32] based on a single theory (item 3). The behavioral theories reported to guide intervention design in the six interventions with a theoretical basis were Social Cognitive Theory (*k* = 2 [25, 26, 29]), Operant Conditioning (*k* = 2 [21, 32]), Multidimensional Theory of Motivation (*k* = 1 [20]), the Health Belief Model (*k* = 1 [19]), the Transtheoretical Model (*k* = 1 study [25, 26]), Information Theory (applied to optimize educational information intake and uptake) (*k* = 1 study [21]), and Cognitive Dissonance Theory (*k* = 1 [21]). Two further interventions [16–18] based their intervention design on the Chronic Care Model, which includes a reference to improving patient self-management via patient activation but is not a behavioral theory per se (i.e., does not propose relationships between predictor variables and behavior). Of the six interventions with a reported theoretical basis, three (50%) [19, 29, 32] mentioned a targeted construct as a predictor of behavior (self-efficacy, motivation, and stimulus-response association, i.e., behavioral reinforcer) and provided evidence to support this link (item 2). The remaining three interventions [20, 21, 25, 26] also mentioned predictors of behavior (self-efficacy [25, 26], motivation [20], and knowledge and skills [21]), but failed to provide supporting evidence linking these constructs to targeted self-management behaviors.

**Table 2:** Application of the Theory Coding Scheme (TCS) to the 17 interventions reviewed

TCS items	Garber (2002), Rintala (2008)*	Guihan (2014)*	Phillips (2002)- Video*	Phillips (2002)-Phone*	Houlihan (2013), Mercier (2015)*	Hossain (2016)*	Worobey (2016)*	Best (2016)*	Ozturk (2011)*	Rowland (2006)*	Rottkamp (1976)*	Scotzin (1990)	Schopp (2007)	Norris (1982)	Phillips (1999)- Video	Phillips (1999)- Phone	Kennedy (2003)	%
1. Theory/model of behavior	Y				Y			Y			Y	Y	Y	Y				41
2. Targeted construct mentioned (with supporting evidence)	Y							Y			Y		Y					24
3. Based on single theory	Y							Y			Y	Y	Y					29
4. Theory used to select recipients																		0
5. Theory used to select/develop intervention techniques					Y			Y			Y	Y	Y	Y				35
6. Theory used to tailor intervention techniques to recipients																		0
7. All intervention techniques explicitly linked to at least one theory-relevant construct																		0
8. At least one, but not all, intervention techniques explicitly linked to at least one theory-relevant construct								Y					Y	Y				18
9. Group of techniques are linked to a group of constructs																		0
10. All theory-relevant constructs explicitly linked to at least one intervention technique																		0
11. At least one, but not all, theory-relevant constructs explicitly linked to at least one intervention technique								Y			Y		Y	Y				24
12. Theory-relevant constructs are measured								Y					Y	Y				18
13. Changes in measured theory-relevant constructs								Y					Y	Y				18
14. Mediational analysis of construct(s)																		0
15. Results discussed in relation to theory																		0
16. Appropriate support for theory																		0
17. Results used to refine theory																		0

*Notes*: Items 13 and 14 of the Theory Coding Scheme (TCS) were not applied in this study. *Y* yes

Interventions marked with an * were tested in randomized controlled trials, the remaining interventions were tested in non-randomized trials

Explanations of how theory guided the design and evaluation of interventions were limited and vague (Table 2, items 4–19).

### Intervention reporting quality

Table 3 presents results of our application of the TIDierR checklist to the 17 interventions reviewed. A summary display of these results is available in Supplementary File 4. None of the intervention descriptions included complete information on all TIDieR items. There were fifteen interventions for which complete information was provided on more than 50% of TIDieR items.

**Table 3:** Application of the Template for Intervention Description and Replication (TIDierR) checklist to the 17 interventions

TIDieR item	Garber (2002), Rintala (2008)*	Guihan (2014)*	Phillips (2002)- Video*	Phillips (2002)-Phone*	Houlihan (2013), Mercier (2015)*	Hossain (2016)*	Worobey (2016)*	Best (2016)*	Ozturk (2011)*	Rowland (2006)*	Rottkamp (1976)*	Scotzin (1990)	Schopp (2007)	Norris (1982)	Phillips (1999)- Video	Phillips (1999)- Phone	Kennedy (2003)	%
1. Brief name	Y	Y	Y	Y	Y	Y	Y	Y	Y	Y	Y	Y	Y	Y	Y	Y	Y	100
2. Rationale	Y	Y			Y	Y		Y		Y	Y	Y	Y		Y	Y	Y	71
3. What: intervention materials																		
3a. For participants	Y		Y	Y	Y				Y		Y		Y	Y	Y			53
3b. For providers							Y	Y	Y					Y			Y	29
4. Intervention procedures	Y					Y	Y	Y	Y	Y	Y		Y	Y	Y	Y	Y	71
5. Who provided the intervention	Y	Y	Y	Y		Y	Y	Y		Y			Y					53
6. How																		
6a. Mode(s) of delivery	Y	Y	Y	Y	Y	Y	Y	Y	Y	Y	Y		Y	Y	Y	Y	Y	94
6b. Individual or group	Y	Y	Y	Y	Y	Y	Y	Y	Y	Y	Y		Y	Y	Y	Y		88
7. Location of intervention	Y	Y	Y	Y	Y	Y	Y	Y	Y	Y	Y			Y	Y	Y	Y	88
8. When and how much																		
8a. No. of sessions	Y	Y	Y	Y	Y	Y	Y	Y	Y	Y	Y	Y	Y	Y	Y	Y		94
8b. Schedule/frequency sessions		Y	Y	Y	Y	Y	Y	Y	Y	Y	Y		Y	Y	Y	Y		82
8c. Duration of sessions	Y		Y	Y	Y		Y	Y	Y	Y	Y		Y					59
9. Tailoring of intervention	Y		Y	Y		Y	Y	Y	Y	Y					Y	Y		59
10. Modifications									Y									6
11. How well (planned)																		
11a. Fidelity assessment methods		Y			Y	Y												18
11b. Fidelity promotion strategies		Y	Y	Y			Y	Y	Y	Y				Y				47
12. Actual fidelity (results)		Y			Y	Y	Y	Y		Y								35

*Note*: *Y* yes, fully reported; empty cells indicate the TIDieR item was rated No, not fully reported

Interventions marked with an * were tested in randomized controlled trials, the remaining interventions were tested in non-randomized trials

The papers reviewed were published between 1976 and 2016. There was no clear pattern in the data suggesting improvements in reporting quality over time. It is noteworthy, however, that the two interventions reported the least comprehensively were published in earlier years (1990 [20] and 2003 [31]), and that the mean proportion of items fully reported on in the 4 interventions reported after 2014 (publication date of the TIDieR guidelines) was slightly higher (74%) than in the 13 studies published on or before this date (59%).

Results suggest that multiple TIDieR items were incompletely reported in the intervention descriptions reviewed, including intervention materials (item 3), who provided the intervention (item 5), duration of sessions (item 8c), tailoring of intervention (item 9), modifications (item 10), fidelity assessment methods (item 11a), presence of fidelity strategies (i.e., development of intervention manuals, training of interventionists, or methods to monitor intervention fidelity; item 11b), and actual intervention fidelity assessment results (item 12). In some cases, efforts were made to report some aspects of the above items, but insufficient information to rate an item as “fully reported” was reported (see decision rules in Supplementary File 2). This was more often the case for the TIDieR items on “who provided the intervention” (item 5) and on “fidelity assessment methods” (item 11a). Several studies reported only the disciplinary background of intervention providers (e.g., nurse, occupational therapist) without specifying additional information on years of experience, number of providers, or specific training provided to deliver the intervention. Similarly, *how* the fidelity assessment was performed may have been indicated (e.g., survey, detailed logs) but details on *who* performed this assessment or kept these records may have been missing. Finally, some interventions as described did not appear to include any form of tailoring (item 9) but this was not explicitly stated by the authors, leaving readers with some degree of uncertainty.

## Discussion

To the best of our knowledge, this study is the first to investigate theory use and quality of reporting in self-management interventions for people with SCI that include a focus on skin care behaviors. Only 6 (35%) of the 17 interventions reviewed were explicitly reported to have a theoretical basis. This is considerably lower than findings from a systematic review of physical activity interventions for people with a physical disability [37] (94% of studies theory-informed). Although there are differences in the methods used to assess theory use in this study, this comparison suggests that theory may currently play a lesser role in SCI skin care self-management research than it does in other areas, despite the applicability of behavioral theories to the field of rehabilitation [38]. In addition, we found that very few explicit links between BCTs and targeted theoretical constructs were articulated compared to a review of behavioral interventions on physical activity and diet [9]. These findings tend to confirm prior observations on the suboptimal reliance on, and reporting of, theory use in intervention design and evaluation in rehabilitation [38]. Although theory-relevant constructs were measured in half of the six theory-based interventions in our analysis, statistical investigations of pathways of change (mediation analyses) were absent (0%), as were discussions of theoretical basis (0%) or suggestions for theory refinements (0%). It is debatable whether empirical research should always include formal theory testing or propose theory refinements, but other reviews of health behavior research have reported higher rates for the above three types of theory use [9, 39]. One explanation for the identified lack of theory use in this study may be a historical tendency within the SCI field to consider interventions focused on prevention of pressure ulcers (or other secondary conditions) from a biomedical rather than a behavioral perspective. Behavioral approaches are certainly warranted when one considers that pressure ulcers are perceived as mostly preventable [3]. Rehabilitation practices targeting prevention typically focus on the adoption and routine performance of skin care behaviors such as regular off-loading of pressure, checking of skin, and appropriate nutrition and fluid intake, among others. A shift to considering behavioral approaches is becoming more evident in recent SCI self-management literature, and may be partially attributed to SCI-specific funding agencies directing funds toward psychosocial research.

In light of our findings, Dunn and colleagues’ recommendations for rehabilitation researchers on theory use and development seem particularly relevant [38]. They include encouragements to (1) identify key variables and the relationships among them to develop a theoretical narrative, (2) link the psychological (cognitions and emotions) with the behavioral, (3) articulate a theory’s prediction clearly and completely, and (4) take small steps toward theory development by embarking on small, manageable empirical projects that test a few, clearly defined propositions, (5) use a combination of quantitative and qualitative methods of inquiry to move toward expanding relevant theories on the basis of logical extensions and results of previous work. Evidence suggests that theory-based interventions result in larger effects, possibly because they are more aligned with the problem and context, or because studies that test them are built with greater care, fidelity, and structure [40]. Researchers who remain unsure of the value in using theory, or for whom use of theory appears daunting, may benefit from Davidoff and colleagues’ work “Demystifying theory and its use in improvement” [10].

Theories reported to have been used in the interventions reviewed in our work included Social Cognitive Theory, the Health Belief Model, and the Transtheoretical Model. These are also the three most commonly used theories in health behavior research [39]. There are over 80 behavioral theories [41], and selecting a theory is a challenging task for intervention designers for whom little guidance is available. More important than using “favorite” theories is the need to select a theoretical basis that is suited to the characteristics of the target population and behavior. Conducting formative research on the factors that influence specific behaviors in a specific population can help selecting a theory. For example, the Theoretical Domains Framework [42] is a tool designed by psychologists and implementation researchers. Researchers should consider its use to inform the design of theory-informed health interventions. It allows for the identification of barriers/facilitators to health behaviors, and the design of tailored and theory-driven interventions. Finally, the validity of any theoretical model should be critically examined prior to use in intervention design. The scientific merit of the Transtheoretical Model for example has been questioned, with some believing it should be abandoned [43].

In terms of intervention reporting quality, our study found high variability in reporting quality across TIDieR items, with complete reporting occurring in 6–100% of interventions. Similar trends to those observed in the current study were found in a review of stroke rehabilitations interventions [44] in which items on intervention and providers, tailoring and modifications, and intervention fidelity were under-reported. It is likely that the nature of some interventions influences the type of information reported in intervention descriptions. For example, a review [45] of supervised exercise training interventions found tailoring to be one of the most well-reported TIDieR items. Compared to self-management interventions, it may be that these interventions are more often delivered to individuals rather than groups, making the personalization component a potentially unique and well-articulated characteristic of the intervention. Authors should aim to report complete information relevant to all TIDieR items, irrespective of intervention type. Where word count is problematic, supplementary materials should be submitted alongside a publication offering a more comprehensive description of the intervention. To report on intervention fidelity, assessment methods need to be considered during study design. Published recommendations [46] to address intervention fidelity in health behavior research can act as a starting point for researchers aiming to include intervention fidelity components in their evaluations.

An interesting finding from our study is that while 6 (35%) interventions were reported to have a theoretical basis, a rationale to support a proposed mechanism of change was mentioned in 12 (71%) interventions. These results are encouraging as they suggest researchers’ reliance on theories of change (i.e., logic models), even though these are not always theory-driven. This is  not necessarily surprising, as a majority of SCI researchers and clinicians are likely to base their study design on a logic model. Explicitly formulating these theories of change and assumptions will improve the evaluation of complex health interventions as they allow the research community to reach a better understanding of how and why a program works [47]. Breuer and colleagues [47] propose a checklist to guide the comprehensive description of theories of change. These recommendations are relevant for rehabilitation researchers designing and reporting intervention research with or without a theoretical basis.

The strengths of this review lies in the reliance on standardized approaches to assess use of theory and intervention reporting quality, and in the involvement of two independent reviewers to apply assessment tools. Eligibility criteria for the review required papers to report the results of an evaluation. Papers describing intervention design and development may have been missed. The effects of this limitation are likely small as references cited in papers and related to tested interventions were consulted. Materials used during intervention delivery (e.g., scripts, PowerPoint slides, educational brochures) were not used in data extraction. Their use may have influenced TIDieR items on intervention content and delivery. This, however, was a deliberate decision, as the TIDieR reporting guidelines aim to improve the descriptions of interventions provided within scientific publications only. Finally, we did not apply the TIDieR tool to control group treatment descriptions. Complete control group treatment descriptions have clinical importance, avoid flawed interpretations of study results, and allow for study replication. Our content analysis of the intervention and control groups included in this review and reported elsewhere suggests that reporting quality for control group treatments was very poor [13].

In conclusion, this study suggests that skin care self-management interventions for people with SCI do to some extent remain “a black box”. A heavier reliance on theory in the design and evaluation of such trials is recommended, and will help reach a better understanding of the mechanisms through which these interventions achieve their effects and the applicability of behavioral theories to this area of work. Similarly, improving the reporting of these interventions will contribute toward building a cumulative and reproducible science of SCI self-management.

## Electronic supplementary material

Search strategies

TiDieR decision rules applied in this study

PRISMA flow diagram

Number and proportion of interventions for which TIDieR items were reported in the 17 interventions reviewed
