# Supplementary material for: Self-management interventions for skin care in people with a spinal cord injury: part 2—a systematic review of use of theory and quality of intervention reporting
Source: Spinal Cord. 2018 May 25;56(9):837–46. doi: 10.1038/s41393-018-0136-5 (PMC6128816; doi:10.1038/s41393-018-0136-5)
Supplement: Supplementary file 2 — TiDieR decision rules applied in this study [file 41393_2018_136_MOESM2_ESM.docx]

**Supplementary Information 2.** TiDieR decision rules applied in this study

| **TIDieR item** | **Explanation in TIDIER checklist**  (adapted from Hoffman et al.*) | **Decision rules and response options** |
| --- | --- | --- |
| Item 1. Brief name: Provide the name or a phrase that describes the intervention | Precision in the name, or brief description, of an intervention enables easy identification of the type of intervention and facilitates linkage to other reports on the same intervention. Give the intervention name, explaining any abbreviations or acronyms in full, or a short (one or two line) statement of the intervention without elaboration. | ‘Yes, fully reported’: authors have provided a short name for the intervention  ‘No, not fully reported’: authors have not provided a short name for the intervention  Note: the short name does not need to be an accurate/comprehensive description of the intervention (*e.g.* referring to telehealth is sufficient) |
| Item 2. Why: Describe any rationale, theory,  or goal of the elements essential to the intervention | Inclusion of the rationale, theory, or goals that underpin an intervention, or the components of a complex intervention, can help others to know which elements are essential, rather than optional or incidental. In some reports, the term “active ingredient” is used and refers to the components within an intervention that can be specifically linked to its effect on outcomes such that, if they were omitted, the intervention would be ineffective. The known or supposed mechanism of action of the active component/s of the intervention should be described. | For this item, do not account for the ‘active essential ingredients’ of the intervention, focus on mechanisms of action/logic model only (see below).  ‘Yes, fully reported’: The mechanism of action/pathway of change of the intervention is described, and authors describe the logic model they have in mind. At minimum, authors have specified the following three logic model components: a) Intervention component – can be far more general than a behaviour change technique (*e.g.* telehealth, education); b) Mediator - mediator of behaviour change or other health outcome; c) Outcome – this can refer to a behavioral outcome or another type of outcome (*e.g.* clinical).  ‘No, not fully reported’: one or more of the three logic model components listed above are not mentioned/unclear.  Note: Educational interventions almost always target knowledge. Knowledge has to be mentioned as a mediator of behaviour change/outcome. In addition, if a theory is mentioned, authors must have specified which constructs from that theory are thought to be part of the mechanism of action (*i.e.* citing the theory alone is not sufficient). |
| Item 3. What (materials): Describe any  physical or informational materials used in  the intervention, including those provided to  participants or used in intervention delivery  or in training of intervention providers.  Provide information on where the materials  can be accessed (for example, online  appendix, URL) | A full description of an intervention should describe what different physical and information materials were used as part of the intervention (this typically will not extend to study consent forms unless they provide written instructions about the intervention that are not provided elsewhere). Intervention materials are the most commonly missing element of intervention descriptions.3 This list of materials can be regarded as comparable with the “ingredients” required for a recipe. It can include materials provided to participants, training materials used with the intervention providers, or the surgical device or pharmaceutical drug used and its manufacturer. For some interventions, it might be possible to describe the materials and the procedures (item 4) together. If the information is too long or complex to describe in the primary paper, alternative options and formats for providing the materials should be used and details of where they can be obtained should be provided in the primary paper. | This item concerns physical and informational materials that are handed over physically to participants or health care professionals. Mention of training does not qualify for this item, unless there are supporting materials distributed during/before/after the training sessions. Use of equipment/devices should be taken into account when considering intervention materials.  This item has been broken down into two sub-items:  3a. For participants  3b. For intervention deliverers   1. ‘Yes, fully reported’ – at a minimum, authors describe some or all of the content in the physical/informational materials (e.g. chapter titles, topics covered). Additional information could include the types of exercises in the materials, the formats involved (e.g. cartoons, pictures, graphs etc). If none of this information is included in the text, but that a URL link or bibliographic reference is provided to access the materials code ‘Yes, fully reported’. 2. ‘No, not fully reported’ – no mention is made of materials or mention is made but none of the required information listed above is provided.   If several materials are mentioned (whether a combination of devices/written materials or several of each) 🡪 only code ‘Yes fully reported,’ if all of them are described using the required minimal information listed above, or all of them are referenced (URL or bibliographic reference in text). If one or more are not described using the minimal information above, then code ‘No, not fully reported’. |
| Item 4. What (procedures): Describe each of the procedures, activities, and/or processes used in the intervention, including any  enabling or support activities | Describe what processes, activities, or procedures the intervention provider/s carried out. Continuing the recipe metaphor used above, this item refers to the “methods” section of a recipe and where intervention materials (“ingredients”) are involved, describes what is to be done with them. “Procedure” can refer to the sequence of steps to be followed and is a term used by some disciplines, particularly surgery, and includes, for example, preoperative assessment, optimisation, type of anaesthesia, and perioperative and postoperative care, along with details of the actual surgical procedure used. Examples of processes or activities include referral, screening, case finding, assessment, education, treatment sessions, telephone contacts, etc. Some interventions, particularly complex ones, might require additional activities to enable or support the intervention to occur (in some disciplines these are known as implementation activities), and these should also be described. Elaboration about how to report interventions where the procedure is not the same for all participants is provided at item 9 (tailoring). | This item considers the level of information and detail provided by authors when describing the intervention delivered and the procedures in place to allow for its delivery. This can relate both to the delivery of the intervention content (*e.g.* details provided on the active ingredients), and to the steps followed to implement the intervention (*e.g.* telehealth implementation in patients’ home, telehealth training provided to participants, any additional person required to deliver the intervention to the patient). This item is not about trial management, monitoring issues, etc.  ’Yes, fully reported’: the description provided by authors must fit the following criteria:   1. I can tell with some confidence what the active ingredients of the interventions were likely to be 2. I can tell with some confidence how to roughly reproduce the intervention 3. I do not have major unanswered questions about how the intervention content was delivered or implemented   ‘No, not fully reported’: the description of the intervention does not match the above criteria  Note: the level of detail required may vary from one study to another, depending on the complexity of the intervention and the amount of additional enabling/supporting activities required. If intervention manuals are provided, and the intervention is not complex, this item is likely to be coded ‘Yes, fully reported’. |
| Item 5. Who provided: For each category of intervention provider (for example,  psychologist, nursing assistant), describe  their expertise, background and any specific training given | The term “intervention provider” refers to who was involved in providing the intervention (for example, by delivering it to recipients or undertaking specific tasks). This is important in circumstances where the providers’ expertise and other characteristics could affect the outcomes of the intervention. Important issues to address in the description might include the number of providers involved in delivering or undertaking the intervention; their disciplinary background (for example, nurse, occupational therapist, colorectal surgeon, expert patient); what pre-existing specific skills, expertise, and experience providers required and if and how these were verified; details of any additional training specific to the intervention that needed to be given to providers before and/or during the study; and if competence in delivering the intervention was assessed before or monitored throughout the study and whether those deemed lacking in competence were excluded or retrained. Other information about providers could include whether the providers were doing the intervention as part of their normal role or were specially recruited as providers for purposes of the study; whether providers were reimbursed for their time or provided with other incentives (if so, what) to deliver the intervention as part of the study, and whether such time or incentives might be needed to replicate the intervention. | ‘Yes, fully reported’: At minimum, the intervention deliverers’ expertise/background (*e.g.* nurse, doctor, social worker) needs to be specified as well as one of the following components: number of providers, pre-existing specific skills relating to intervention delivery, training provided to them. Authors stating that an ‘experienced nurse’ delivered the intervention is not sufficient.  ‘No, not fully reported’: the minimum criteria listed above are not described in the paper. |
| Item 6. How: Describe the modes of delivery (such as face to face or by some other mechanism, such as internet or telephone)  of the intervention and whether it was provided individually or in a group | Specify whether the intervention was provided to one participant at a time (such as a surgical intervention) or to a group of participants and, if so, the group size. Also describe whether it was delivered face to face, by distance (such as by telephone, surface mail, email, internet, DVD, mass media campaign, etc), or a combination of modes. When relevant, describe who initiated the contact, and whether the session was interactive or not,  and any other delivery features considered essential or likely to influence outcome. | This item was broken down into two sub-items:  6a. Mode of delivery (*e.g.* Face to face or by some other mechanism, such as internet or telephone)  6b. Individual versus group delivery  ‘Yes, fully reported’ if this information is clearly reported  ‘No, not fully reported’ if this information is incomplete. |
| Item 7. Where: Describe the type(s) of  location(s) where the intervention occurred,  including any necessary infrastructure or relevant features | In some studies the intervention can be delivered in the same location where participants were recruited and/or data were collected and details might therefore already be included in the primary paper (for example, as in item 4b of CONSORT 2010 statement if reporting a trial). If, however, the  intervention occurred in different locations, this should be specified. At its simplest level, the location might be, for example, in the participants’ home, residential aged care facility, school, outpatient clinic, inpatient hospital room, or a combination of locations. Features or circumstances about the location can be relevant to the delivery of the intervention and should be described. For example, they might include the country, type of hospital or primary care, publicly or privately funded care, volume of activity, details of the healthcare system, or the availability of certain facilities or equipment. These features can impact on various aspects of the intervention such as its feasibility or provider or participant adherence and are important for those considering replicating the intervention. | ‘Yes, fully reported’: The authors should have made clear where the intervention was delivered (participants’ home, in rehab center, in day hospital, etc.)  ‘No, not fully reported’: it remains unclear where the intervention was delivered. |
| Item 8. When and how much: Describe the  number of times the intervention was delivered and over what period of time  including the number of sessions, their  schedule, and their duration, intensity or dose | The type of information needed about the “when and how much” of the intervention will differ according to the type of intervention. For some interventions some aspects will be more important than others. For many non pharmacological interventions, the “how much” of the intervention is instead described by the duration and number of sessions. For multiple session interventions, the schedule of the sessions is also needed and if the number of sessions, their schedule, and/or intensity was fixed or if it could be varied according to rules and if so, what they were. | This item was broken down into three sub-items:  8a. One item on ‘Number of sessions’  8b. One item on ‘Schedule/Frequency of sessions’  8c. One item on ‘Duration of each session’  A sub-item on ‘intensity/dose was considered as per the TIDieR recommendations, but was found not to apply to the interventions in this review and was therefore removed.  ‘Yes, fully reported’: the information for the sub-item is clear in the paper. If there are several components to the intervention (*e.g.* group sessions supplemented with phone calls), the above information needs to be reported for each component of the intervention for ‘Yes, fully reported’ to be coded. If intervention is delivered in one session only, code item 8b on schedule/frequency as ‘yes, fully reported’.  ‘No, not fully reported’: the information for the sub-item is not clear in the paper. |
| Item 9. Tailoring: If the intervention was  planned to be personalized, titrated or  adapted, then describe what, why, when, and  how | In tailored interventions, not all participants receive an identical intervention. Interventions can be tailored for several reasons, such as titration to obtain an appropriate “dose”; participant’s preference, skills, or situation; or it may be an intrinsic element of the intervention as with increasing intensity of an exercise. Tailoring can occur at several stages and authors should describe any decision points and rules used at each point . If any decisional or instructional materials are used, such as flowcharts, algorithms etc., these should be included, referenced, or their location provided. | Tailored interventions are those designed to address the individual characteristics of persons within a sample, such as personality factors, goals, needs, preferences, and resources**. They are not restricted to interventions where tailoring is more systematic and controlled (*e.g.* people with baseline scores < x are given one educational messages that are different from those with a baseline score > x.  ‘Yes, fully reported’: Some tailoring is reported and sufficient details are provided on the what, why, when and how it was designed/implemented.  ‘No, not fully reported’: No mention made of whether tailoring was a component of the intervention, or some tailoring is mentioned but insufficient details are provided on the what, why, when, and how. |
| Item 10. Modifications: If the intervention was  modified during the course of the study, describe the changes (what, why, when, and  how) | This item refers to modifications that occur at the study level, not individual tailoring as described in item 9. Unforeseen modifications to the intervention can occur during the course of the study, particularly in early studies. If this happens, it is important to explain what was modified, why and when modifications occurred, and how the modified intervention differed from the original (*e.g.* modification to who provided the intervention, modification in the materials). Modifications sometimes reflect changing circumstances. In other studies, they can show learning about the intervention, which is important to transmit to the reader and others to prevent unnecessary repetition of errors during attempts to replicate the intervention. If changes to the intervention occurred between the published protocol or published pilot study and the primary paper, these changes should also be described. | Modifications can apply both to the intervention content and intervention-related procedures.  ‘Yes, fully reported’: Some modifications reported and sufficient details are provided.  ‘No, not fully reported’: No mention is made of whether modifications were made, or some modifications are reported but authors have not provided sufficient detail. |
| Item 11. How well (planned): If intervention  adherence or fidelity was assessed, describe  how and by whom, and if any strategies were  used to maintain or improve fidelity, describe  them | Fidelity refers to the degree to which an  intervention happened in the way the investigators intended it to and can affect the success of an intervention. The terms used to describe this concept vary among disciplines and include treatment integrity, provider or participant adherence, and implementation fidelity. This item—and item 12—extends beyond simple receipt of the intervention (such as how many participants were issued with the intervention drug or exercises) and refers to “how well” the intervention was received or delivered (such as how many participants took the drug/did the exercises, how much they took/did, and for how long). Depending on the intervention, fidelity can apply to one or more parts of the intervention, such as training of providers, delivery of the intervention, and receipt of the intervention. The types of measures used to determine intervention fidelity will also vary according to the type of intervention. | This item is broken down into two sub-items:  11a. Intervention fidelity assessment methods  11b. Presence of fidelity strategies  11a. Intervention fidelity assessment methods  ‘Yes, fully reported’: Some assessment of intervention fidelity/adherence to protocol is mentioned and sufficient details are provided on how/by whom.  ‘No, not fully reported’: No mention is made of whether intervention fidelity was assessed, or some intervention fidelity/adherence to protocol measure is mentioned, but insufficient details are provided on how/by whom the assessment is carried out.  Note: authors do not have to refer to fidelity/adherence to protocol specifically. Any measure that reviewers believe is an indicator of intervention fidelity (*e.g.* delivery, receipt etc) can be considered part of an assessment of fidelity, regardless of the terminology used.  11b. Presence of fidelity strategies  Authors should ideally report on the inclusion in the study design of strategies to ensure that the intervention is delivered according to plan. The following occurrences count as fidelity strategies^***^  (whether referred to using this terminology or not):   1. *Developing manuals* that spell out the program purpose, goals, session objectives, and essential or critical elements, such as behaviors that are to be role modeled by interventionists or the content that must be covered, can standardize the training of interventionists 2. *Training of the interventionists* - Research team members bring a wide range of skills and experiences to a study and this can influence the manner in which the intervention is delivered. The study planners must consider the interventionists' skill sets — what they bring to the project. Strategies for achieving conformity across multiple interventionists include training for intervention delivery in which (a) the philosophy of the program is discussed, (b) objectives and intervention procedures are covered in detail, and (c) the interventionists have an opportunity to practice the necessary skill sets needed for the intervention. 3. *Monitoring of intervention fidelity* to make corrections as appropriate, e.g. observations of intervention sessions by an observer, possibly with feedback to intervention deliverers when appropriate.   ‘Yes, fully reported’: it is clear whether one or more of the above fidelity strategies have been used to ensure the intervention is delivered as planned.  ‘No, not fully reported’: none of the above fidelity strategies are mentioned (note: the level of detail provided on these fidelity strategies is irrelevant for this item, we are looking for their presence/absence in the intervention/study description). |
| Item 12: How well (actual): If intervention  adherence or fidelity was assessed, describe the extent to which the intervention was delivered as planned | For various reasons, an intervention, or parts of it, might not be delivered as intended, thus affecting the fidelity of the intervention. If this is assessed, authors should describe the extent to which the delivered intervention varied from the  intended intervention. This information can help to explain study findings, minimise errors in interpreting study outcomes, inform future modifications to the intervention, and, when  fidelity is poor, can point to the need for further studies or strategies to improve fidelity or adherence | ‘Yes, fully reported’: data collected for the intervention fidelity/adherence to protocol measures (item 11) are reported.  ‘No, not fully reported’: data on intervention fidelity/adherence to protocol measures are not reported in sufficient detail for readers to conclude whether the intervention fidelity aspects measured were generally delivered as intended, or intervention fidelity/adherence to protocol not reported to have been assessed (see response to item 11). |

* Hoffman et al. (2015). What’s in a name? The challenge of describing interventions in systematic reviews: analysis of a random sample of reviews of non-pharmacological stroke interventions. *BMJ Open*, 5 (11).

**definition taken from Beck et al (2010) Challenges in Tailored Intervention Research, *Nurs Outlook*, 58(2).

^***^ definition taken from Horner et al (2006)- Enhancing Intervention Fidelity: A Means of Strengthening Study Impact. *J Spec Pediatr Nurs*. 11(2).
