# Supplementary figures and images for: Self-management interventions for skin care in people with a spinal cord injury: part 2—a systematic review of use of theory and quality of intervention reporting
Source: Spinal Cord. 2018 May 25;56(9):837–46. doi: 10.1038/s41393-018-0136-5 (PMC6128816; doi:10.1038/s41393-018-0136-5)

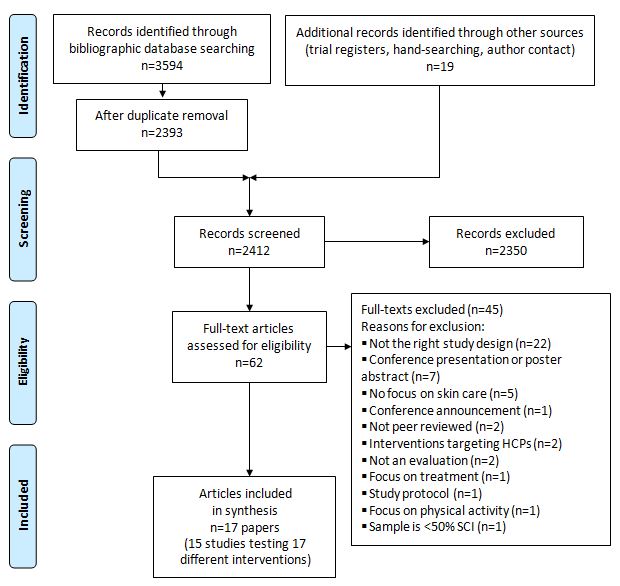

Supplement: Supplementary file 3 — PRISMA flow diagram [file 41393_2018_136_MOESM3_ESM.jpg]

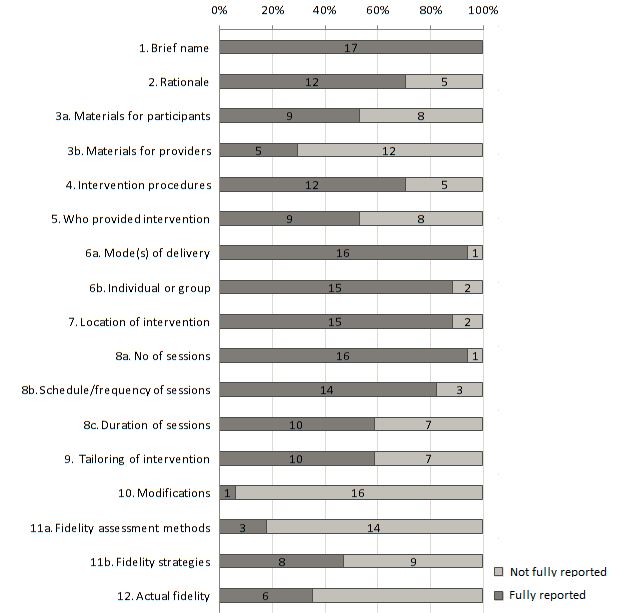

Supplement: Supplementary file 4 — Number and proportion of interventions for which TIDieR items were reported in the 17 interventions reviewed [file 41393_2018_136_MOESM4_ESM.jpg]
